# Safety and Efficacy of a Small J-Tipped Guidewire for Pancreatic Duct Endoscopic Intervention

**DOI:** 10.1155/2019/8947614

**Published:** 2019-04-01

**Authors:** Sumio Hirose, Mitsuharu Fukasawa, Shinichi Takano, Makoto Kadokura, Hiroko Shindo, Ei Takahashi, Yudai Yokota, Yoshimitsu Fukasawa, Satoshi Kawakami, Hiroshi Hayakawa, Tadashi Sato, Nobuyuki Enomoto

**Affiliations:** First Department of Internal Medicine, Faculty of Medicine, University of Yamanashi, Yamanashi 409-3898, Japan

## Abstract

**Background and Aims:**

The insertion of the guidewires (GWs) into the pancreatic duct is technically difficult, and there is a risk of post-ERCP pancreatitis (PEP). The aim of this study was to evaluate the safety and efficacy of a small J-tipped guidewire for pancreatic duct endoscopic intervention.

**Methods:**

This single-site retrospective study was conducted to assess the procedural success rate and adverse events of endoscopic transpapillary interventions to the pancreatic duct in 114 cases using the small J-tipped GW and 180 cases using the angle-tipped GW.

**Results:**

The procedural success rate was significantly higher in the small J-tipped GW group compared with that in the angle-tipped GW group (76% versus 47%, P < 0.001). The procedural success-related factors were chronic pancreatitis (OR 0.43, 95% CI 0.22–0.82, P = 0.01), flexion angle of the pancreatic duct < 90° (OR 0.50, 95% CI 0.30–0.80, P = 0.01), and use of the small J-tipped GW (OR 4.63, 95% CI 2.61–8.20, P < 0.001). The rates of total post-ERCP pancreatitis were significantly lower in the small J-tipped GW group compared with that in the angle-tipped GW group (3.5% versus 12.2%, P = 0.01). Multivariate analysis of pancreatitis risk factors indicated that only the use of the small J-tipped GW was a factor in decreasing the risk of developing pancreatitis (OR 0.12, 95% CI 0.09–0.85, P = 0.02).

**Conclusions:**

Small J-tipped GWs increase the success rate of the pancreatic duct endoscopic intervention as well as a reduced risk of developing postoperative pancreatitis.

## 1. Introduction

Endoscopic transpapillary interventions to the pancreatic duct (pancreatic duct endoscopic intervention), such as a detailed examination for pancreatic diseases and stenting for pancreatic duct stricture, became more common in recent years.

Although pancreatic duct endoscopic intervention is usually performed with guidewire (GW) placed in the pancreatic duct, the insertion of a GW itself is difficult because the pancreatic duct is anatomically more complicated with advanced curves and strictures compared with the biliary duct [1]. In addition, GW insertion into the pancreatic duct is reportedly a risk factor for post-ERCP pancreatitis (PEP), causing papillary edema and injury of the pancreatic duct and parenchyma [2–6].

Pancreatic stent placement has been reported to be beneficial in preventing PEP because it prevents pancreatic outflow obstruction due to papillary edema [7–10]. However, pancreatitis due to pancreatic injury cannot be controlled even with the use of a pancreatic stent. We have previously reported that ERCP with a GW inserted into the pancreatic duct results in a significantly higher incidence of PEP compared with procedures without a GWinsertion into the pancreatic duct, regardless of the use of a pancreatic stent [11]. Improvement of the GW, increasing insertability and preventing the injury of the pancreatic duct, is necessary.

Recently, J-tipped GW designed for improving luminal passage has been developed and reported to be beneficial in selective biliary cannulations [12, 13]. Although J-tipped GW was not applicable to pancreatic duct endoscopic intervention due to its large tip radius, the newly developed small J-tipped GW with a smaller loop has enabled pancreatic interventions. In this study, we assessed the safety and efficacy of a small J-tipped guidewire for pancreatic duct endoscopic intervention.

## 2. Materials and Methods

### 2.1. Participants

This study was conducted at the University of Yamanashi Hospital between January 2011 and September 2016. All patients underwent endoscopic transpapillary intervention with the GW insertion into the pancreatic duct for pancreatic disease or pancreatic GW technique (a method for selective biliary cannulation by stabilizing the duodenal papilla using a GW indwelling in the pancreatic duct). We used angle-tipped GWs from January 2011 to February 2014 and small J-tipped GWs from March 2014 to September 2016. The patients were divided into two groups according to treatment timing based on the type of GW used: the angle-tipped GW group (from January 2011 to February 2014) and the small J-tipped GW group (from March 2014 to September 2016).

The following cases were excluded and only the first treatment was analyzed for overlapping cases:

1. Difficulty in the endoscopic approach (esophageal stricture, gastric outlet obstruction, and duodenal stenosis)
2. Previous gastrectomy with Billroth II or Roux-en-Y reconstruction
3. Previous papillectomy
4. Approach from the minor papilla
5. Precutting
6. Recent history of acute pancreatitis
7. Use of other types of GWs for selective pancreatic cannulation instead of small J-tipped or angle-tipped GWs
8. Use of Diclofenac

### 2.2. Study Design

This is a retrospective observational study at a single center.

### 2.3. GWs

**Figure 1:**
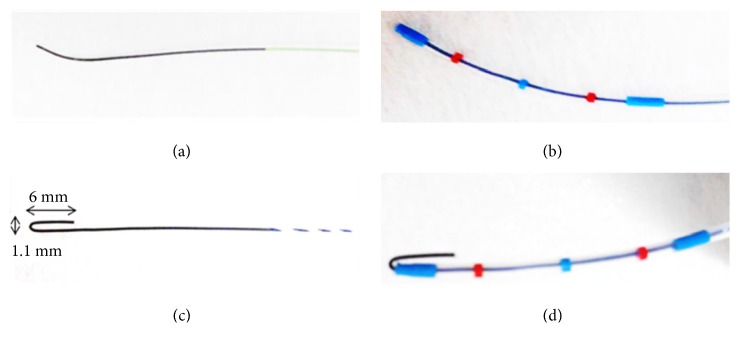
Guidewires. (a) Angle-tipped guidewire. (b) Neutral positions of angle-tipped guidewire. (c) Small J-tipped guidewire. The tip was bent to attain a 0.55-mm radius. (d) Neutral positions of small J-tipped guidewire.

Angle-tipped GWs (VisiGlideTM, 0.025-inch, Olympus, Tokyo, Japan) or small J-tipped GWs (RWHJ-2545SJ, 0.025-inch; Paiolax Medical Devices, Inc., Kanagawa, Japan) were used for insertion into the pancreatic duct (first GW) (Figure 1). Both types have a soft tip made of hydrophilic material with a rigid shaft comprising a sheath and a jacket. The tip of angle-tipped GW was bent at 30°, and that of small J-tipped GW was bent to attain a diameter of 1.1 mm. If deep GW passage with first GW was difficult, the GW was changed to high-torque, flexible, hydrophilic GWs (NaviProTM, 0.035-inch, Boston scientific, MA, USA) as second GW. The second GW has a flexible structure only for insertion and does not have the rigidity to conduct interventions after insertion. Therefore, the second GW was replaced with the first GW for intervention once it reached the target site.

### 2.4. Endoscopic Procedures

The procedures were carried out using side-viewing duodenoscopes (JF260V, Olympus, Tokyo, Japan) and a standard injection catheter (MTW Endoscopie, Dusseldorf, Germany). Neutral positions of each GWs for cannulation to the pancreatic duct were determined as follows: the angle-tipped GW was used aligned with the tip of catheter, and the small J-tipped GW was used with the J shape of the tip (Figure 1).

Selective pancreatic cannulation was conducted using the contrast method. All endoscopic procedures were performed by dedicated ERCP training fellow under directly supervising senior endoscopists who had more than 1000 ERCP experiences. Eight trainees in the angle-tipped GW group and 10 trainees in the small J-tipped GW group performed endoscopic intervention, but the 2 trainers who supervising them were common in both groups.

All procedures were performed under conscious sedation using intravenous midazolam (3–10 mg) and pentazocine (15 mg). Scopolamine butylbromide (20 mg) or glucagon (1 mg) was intravenously administered to control the intestinal peristaltic motion. To prevent PEP, ulinastatin (50,000 U/day) was intravenously administered immediately after treatment.

### 2.5. Outcome Measurements

*(1) Assessment of Efficacy*. The first GW procedure completion rate and final procedure completion rate were compared between the small J-tipped and angle-tipped GW groups. An analysis was also conducted on factors related to the first GW procedure completion.

*(2) Assessment of Safety*. The post-ERCP complication rate and severity were compared between the small J-tipped and angle-tipped GW groups. A diagnosis of PEP was made on the presence of continuous abdominal pain at least 24 h after procedure and an increase in serum amylase level greater than three times the upper normal limit. Severity was classified according to the modified Cotton's criteria [14] with the duration of hospitalization replaced with that of fasting4. PEP requiring an extension of fasting for 1–3 days was defined as mild, with fasting for 4–10 days defined as moderate and that for 10 days or more, followed by necrosis or pseudocyst, or requiring drainage or surgery defined as severe.

An analysis was also performed pertaining to the risk factors for PEP.

### 2.6. Statistical Analysis

The chi-squared test or the Fisher's exact test was used for a univariate analysis of category data, with the Student's t-test used for the analysis of quantitative data. In assessing the first GW procedure completion-related factors and PEP risk factors, a multivariate analysis was performed using multiple logistic regression analysis, which was conducted after univariate analysis using factor P < 0.20. The level of statistical significance was set at P < 0.05.

## 3. Results

### 3.1. Participants

**Figure 2:**
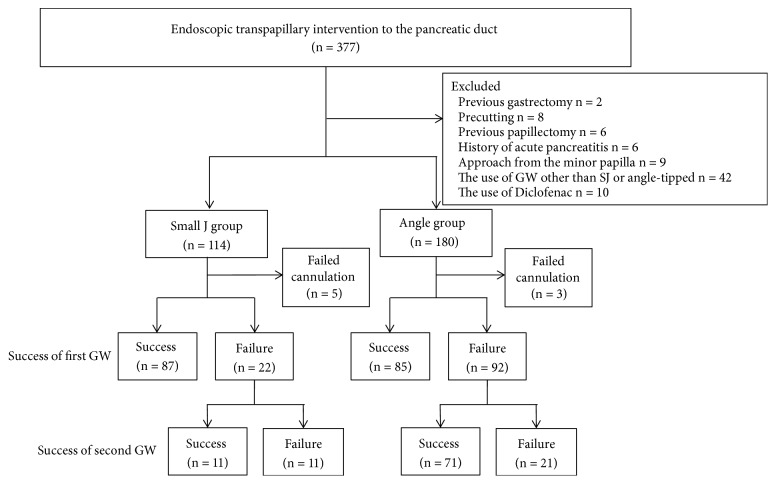
Participant flow, including clinical courses in this study.

In total, 377 patients underwent pancreatic duct endoscopic intervention between January 2011 and September 2016. After excluding patients who met any of the exclusion criteria, we enrolled 114 patients in the small J group and 180 patients in the angle group (Figure 2).

Table 1 shows the patient backgrounds of both groups. The mean age of patients in the small J group, which included 43 females (38%), was 69 years (19–89 years). The mean age of patients in the angle group, which included 79 females (44%), was 72 years (33–90 years). The diseases in small J and angle groups were pancreatic cancer in 11 (10%) and 36 (20%) cases, pancreatic cystic tumor in 41 (36%) and 53 (30%) cases, chronic pancreatitis in 31 (27%) and 25 (14%) cases, and biliary disease in 25 (22%) and 62 (34%) cases, respectively (difficult biliary cannulation cases in which insertion into the biliary duct was performed with a GW indwelling in the pancreatic duct). The small J group included pancreatic cancer and biliary disease significantly less frequently (10% versus 20%, P = 0.02, 22% versus 34%, P = 0.03, respectively) and chronic pancreatitis significantly more frequently (27% versus 14%, P = 0.006) compared with the angle-tipped GW group. According to the pancreatic duct findings, the small J-tipped GW group had significantly more cases with flexion angle of the pancreatic duct < 90° than the angle-tipped GW group (52% versus 39%, P = 0.04).

### 3.2. Procedural Success Rates with First GW

Selective pancreatic cannulation succeeded in 109 (96%) cases in the small J-tipped GW group and 177 (98%) cases in the angle-tipped GW group (Figure 2).  Table 2 shows the procedural completion rates. The first GW procedure success rate was significantly higher in the small J group (76%) than that in the angle group (47%) (P < 0.001). On the other hand, there were no significant differences between the groups in the overall success rate of procedures when a second GW was also used (P = 0.86).

**Figure 3:**
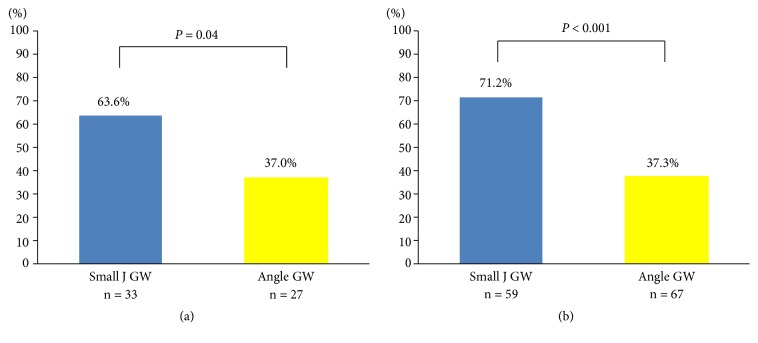
The rate of procedure completion by first guidewire. (a) Patients with chronic pancreatitis. (b) Patients with flexion angle of the pancreatic duct < 90°.

Table 3 shows the results of the analysis of first GW procedure success-related factors. These significant factors were chronic pancreatitis (OR 0.43, 95% CI 0.22–0.82, P = 0.01), flexion angle < 90° (OR 0.50, 95% CI 0.30–0.85, P = 0.01), and the use of a small J-tipped GW (OR 4.63, 95% CI 2.61–8.20, P < 0.001). The first GW procedure success rate in cases with difficult factors in small J and angle groups were 63.6% and 37.0% with chronic pancreatitis and 71.2% and 37.3% with a flexion angle < 90°, respectively; the small J group showed significantly higher success rates for both factors (P = 0.04; P < 0.001) (Figure 3).

### 3.3. Adverse Events

Table 4 shows adverse events observed in this study, all of which were PEP. PEP incidence was significantly lower in the small J group (3.5%) than that in the angle group (12.2%) (P = 0.01). The details of severity in small J and angle groups were 0 and 3 (1.7%) severe cases, 1 (0.9%) and 7 (3.9%) moderate cases, and 3 (2.6%) and 12 (6.6%) mild cases, respectively.

Table 5 shows the results of the analysis of risk factors for PEP. Only the use of the small J-tipped GW was identified as a factor causing a decrease in the risk of developing PEP (OR 0.12, 95% CI 0.09–0.85, P = 0.02). Despite the observation of significant differences between pancreatic cancer, biliary disease; and chronic pancreatitis as background diseases in both groups, these diseases were not significant factors for the development of PEP.

## 4. Discussion

Although pancreatic duct endoscopic intervention is performed with a GW indwelling in the pancreatic duct, the insertion of the GW is difficult because the pancreatic duct is anatomically complicated with advanced curves and strictures. In recent years, J-tipped GWs to be used for luminal passage and stricture penetration have been developed and reported to be beneficial in selective biliary cannulation. Although the J-shaped tip is considered to be more suitable for pancreatic intervention owing to the low risks of damaging the pancreatic duct and wrong insertion into a branch duct, the utility and safety of J-shaped tip GW in pancreatic intervention had not previously been examined [12]. Here, the initial procedure success rate with the small J-tipped GW was significantly higher than that with the angle-tipped GW. Moreover, according to the results of the analysis of the first GW procedure success-related factors, including patient background, factors of the pancreatic duct, and procedural factors, the use of the small J-tipped GW was identified as an independent factor for procedural success and achieved favorable outcomes even in cases with difficult factors such as chronic pancreatitis or strong flexion angle in the pancreatic duct. Small J-tipped GWs with a loop-shaped tip allowed for insertion along the main pancreatic duct without any unintended insertion into branches and were able to pass through smoothly without colliding with anything despite extremely tight flexion angles, which was impossible for an angle-tipped GW. Hydrophilic GWs, used for second GW in this study, have a better insertability due to their flexibility and high-torque transmissibility; the drawback is their low supportability with a soft shaft. When a hydrophilic GW is used as the first GW, it needs to be exchanged to a more supportive GW with a stiff shaft; once it reaches the target site, the treatment inevitably requires two GWs. This not only results in a disadvantage from the aspect of cost but also causes a complexity in the procedure and an extension of treatment time. Meanwhile, small J-tipped GWs are deemed to be beneficial in pancreatic duct endoscopic intervention, with sufficient insertability and supportability to complete the procedure using them alone.

The major problem associated with pancreatic duct endoscopic intervention is postoperative pancreatitis. GW insertion into the pancreatic duct is considered to be a risk factor for pancreatitis and injury of the pancreatic duct caused by the GW may result in serious outcomes [2–6, 11]. Although stent placement in the pancreatic duct is reported to be beneficial for prevention of the incidence of PEP caused by duodenal papilledema [7–10], the preventive effect against PEP in cases with injury of the pancreatic duct has not been confirmed. GWs need to be improved to prevent injury of the pancreatic duct.

Sakai et al. have reported on the preventive effect of loop-tipped GWs against PEP [6]. Loop-tipped GWs resulted in less frequent unintended insertion of the guidewire into a side branch of the pancreatic duct than conventional GWs and led to significantly lower serum amylase levels after treatment. However, there was no difference in PEP incidence. The small sample size in the study (n = 20) was regarded as a limitation and the necessity of a large-scale study was suggested. In our study, we assessed the preventive effect of small J-tipped GWs against PEP on 114 cases. Results suggested that the use of the small J-tipped GW caused significantly less frequent PEP than that of the angle-tipped GW. This is probably because the tip of a small J-tipped GW is loop shaped and has a low risk of damaging the pancreatic duct. Accordingly, this type of GW has a safe structure for use in pancreatic interventions.

This study had several limitations. First, it had a retrospective design and there were intergroup differences in background diseases and pancreatic duct findings (the small J-tipped GW group had significantly more cases with chronic pancreatitis and a flexion angle < 90° and the angle-tipped GW group had more cases with pancreatic cancer and biliary disease). Although these factors may have affected efficacy and safety as bias factors, it is evident that the utility of small J-tipped GWs is high because chronic pancreatitis and acute-angled pancreatic duct are rather difficult procedure-related factors [1]. In addition, the results of the analysis of complication-related factors showed that these factors were not significant. Second, Urinastatin was used for prevention of PEP in this study. Although indomethacin is the standard treatment for prevention of PEP, a 100-mg rectal dose which showed the efficacy in the previous reports exceeds the standard dose of Japanese patients. Ulinastatin was not a standard treatment, but since it was administered equally to both groups, it does not affect the result.

In conclusion, present study suggested that small J-tipped GWs not only offered better usefulness than angle-tipped GWs in pancreatic duct endoscopic intervention but also reduce the risk of PEP.

## 5. Conclusions

Small J-tipped GWs increase the success rate of the pancreatic intervention as well as a reduced risk of developing postoperative pancreatitis.

## Figures and Tables

**Table 1:** Characteristics.

	Small J GW	Angle GW	*P *value
	(n = 114)	(n = 180)	
*Patients characteristics*			
Age, median (range), y	69 (19–89)	72 (33–90)	0.17
Female sex, n (%)	43 (38)	79 (44)	0.24
Disease, n (%)			
Pancreatic cancer	11 (10)	36 (20)	0.02
Pancreatic cystic tumor	41 (36)	53 (30)	0.25
Chronic pancreatitis	31 (27)	25 (14)	0.006
Biliary disease	25 (22)	62 (34)	0.03
Others	6 (5)	4 (2)	0.19
*Findings of pancreatic duct*			
Flexion angle < 90°, n (%)	59 (52)	70 (39)	0.04
Stenosis, n (%)	23 (20)	34 (19)	0.88

EPS, endoscopic pancreatic stent; ENPD, endoscopic nasopancreatic drainage.

**Table 2:** Success rate of endoscopic procedure.

	Small J GW	Angle GW	*P* value
	(n = 114)	(n = 180)	
*Procedure completion rate*			
First GW, n (%)	87 (76)	85 (47)	< 0.001
Overall, n (%)	98 (86)	156 (87)	0.86

GW, guidewire.

**Table 3:** Univariate and multivariate analysis of factors for procedure completion.

	Univariate			Multivariate		
Variable	OR	95%CI	*P *value	OR	95%CI	*P* value
*Patient factor*						
Age < 60y	0.86	0.47 - 1.57	0.62			
Female	0.60	0.38 - 0.97	0.04	0.61	0.37 - 1.02	0.06
Pancreatic cancer	0.99	0.52 - 1.88	0.98			
Pancreatic cystic tumor	1.13	0.68 - 1.87	0.63			
Chronic pancreatitis	0.66	0.38 - 1.17	0.15	0.43	0.22 - 0.82	0.01
Biliary disease	0.88	0.53 - 1.47	0.63			
*PD findings*						
Flexion angle < 90°	0.64	0.40 - 1.03	0.07	0.50	0.30 - 0.85	0.01
Stenosis	0.89	0.49 - 1.63	0.71			
*Guidewire*						
Small J	3.52	2.09 - 5.93	< 0.001	4.63	2.61 - 8.20	< 0.001

CI, confidence interval; PD, pancreatic duct; GW, guidewire.

**Table 4:** The rate of complication.

	Small J GW	Angle GW	*P* value
	(n = 114)	(n = 180)	
*PEP, n (*%)	4 (3.5)	22 (12.2)	0.01
Severe	0	3 (1.7)	
Moderate	1 (0.9)	7 (3.9)	
Mild	3 (2.6)	12 (6.6)	
*Hemorrhage*	0	0	1.00
*Perforation*	0	0	1.00

PEP, post-ERCP pancreatitis.

**Table 5:** Univariate and multivariate analysis of risk factors for post-ERCP pancreatitis.

	Univariate			Multivariate		
Variable	OR	95%CI	*P *value	OR	95%CI	*P* value
*Patient factor*						
Age < 60y	1.83	0.72 - 4.60	0.21			
Female	1.73	0.77 - 3.88	0.18	1.57	0.69 - 3.57	0.28
Pancreatic cancer	0.98	0.32 - 2.98	0.97			
Pancreatic cystic tumor	1.18	0.51 - 2.76	0.70			
Chronic pancreatitis	1.16	0.45 - 3.03	0.76			
Biliary disease	0.93	0.38 - 2.30	0.88			
*Intervention*						
Cunnulation time > 10 min	1.29	0.57 - 2.93	0.54			
Endoscopic sphincterotomy	0.36	0.05 - 2.74	0.32			
Intraductal ultrasonography	0.44	0.13 - 1.52	0.20	0.56	0.16 - 1.96	0.36
Blushing cytology	1.22	0.47 - 3.18	0.69			
Pancreatic drainage						
EPS	0.64	0.26 - 1.57	0.33			
ENPD	1.02	0.45 - 2.27	0.97			
*Guidewire*						
Small J	0.26	0.09 - 0.78	0.02	0.12	0.09 - 0.85	0.02
*PD findings*						
Flexion angle < 90°	0.96	0.43 - 2.17	0.92			
Stenosis	1.09	0.39 - 3.04	0.87			

CI, confidence interval; EPS, endoscopic pancreatic stenting; ENPD, endoscopic nasopancreatic drainage; PD, pancreatic duct; GW, guidewire.

## Data Availability

No data were used to support this study.

## Supplementary Materials

Supplementary 1 Video 1: insertion into pancreatic duct with angle-tip guidewire.

Supplementary 2 Video 2: insertion into pancreatic duct with small J-tipped guidewire.
